# Severe Housing Insecurity during Pregnancy: Association with Adverse Birth and Infant Outcomes

**DOI:** 10.3390/ijerph17228659

**Published:** 2020-11-21

**Authors:** Kathryn M. Leifheit, Gabriel L. Schwartz, Craig E. Pollack, Kathryn J. Edin, Maureen M. Black, Jacky M. Jennings, Keri N. Althoff

**Affiliations:** 1 Department of Health Policy and Management, Los Angeles Fielding School of Public Health, University of California, Los Angeles, CA 90095, USA; 2 Department of Epidemiology, Johns Hopkins Bloomberg School of Public Health, Baltimore, MD 21205, USA; cpollac2@jhmi.edu (C.E.P.); Jennings@jhmi.edu (J.M.J.); kalthoff@jhu.edu (K.N.A.); 3 Center for Child and Community Health Research, Department of Pediatrics, Johns Hopkins University School of Medicine, Baltimore, MD 21224, USA; 4 Institute for Health Policy Studies, University of California, San Francisco School of Medicine, San Francisco, CA 94118, USA; gabriel.schwartz2@ucsf.edu; 5 Department of Social & Behavioral Sciences, Harvard T.H. Chan School of Public Health, Boston, MA 02115, USA; 6 Department of Health Policy and Management, Johns Hopkins Bloomberg School of Public Health, Baltimore, MD 21205, USA; 7 Department of Medicine, Johns Hopkins University School of Medicine, Baltimore, MD 21205, USA; 8 School of Nursing, Johns Hopkins University, Baltimore, MD 21205, USA; 9 Department of Sociology, Princeton University, Princeton, NJ 08544, USA; kedin@princeton.edu; 10 School of Public and International Affairs, Princeton University, Princeton, NJ 08544, USA; 11 Department of Pediatrics, University of Maryland School of Medicine, Baltimore, MD 21201, USA; mblack@som.umaryland.edu; 12 RTI International, Research Triangle Park, NC 27709, USA

**Keywords:** housing, eviction, homeless persons, birth weight, premature birth, neonatal intensive care units, infant health

## Abstract

**Introduction:** Housing insecurity is increasingly commonplace among disadvantaged women and children. We measured the individual- and population-level impact of severe housing insecurity during pregnancy on adverse birth and infant outcomes. **Methods:** We analyzed data from 3428 mother–infant dyads enrolled in the Fragile Families and Child Wellbeing Study, a prospective cohort study representing births in 20 large U.S. cities from 1998 to 2000. Severe housing insecurity was defined as threatened eviction or homelessness during pregnancy. Outcomes included low birth weight and/or preterm birth, admission to a neonatal intensive care unit (NICU) or stepdown facility, extended hospitalization after delivery, and infant health and temperament. We estimated exposure–outcome associations with risk ratios adjusted for pre-pregnancy maternal sociodemographic and heath factors and calculated a population attributable fraction (PAF) of outcomes attributable to severe housing insecurity. **Results:** We found statistically significant associations between severe housing insecurity during pregnancy and low birth weight and/or preterm birth (risk ratio (RR] 1.73, 95% confidence interval (CI) 1.28, 2.32), NICU or stepdown stay (RR 1.64, CI 1.17, 2.31), and extended hospitalization (RR 1.66, CI 1.28, 2.16). Associations between housing insecurity and infant fair or poor health (RR 2.62, CI 0.91, 7.48) and poor temperament (RR 1.52, CI 0.98, 2.34) were not statistically significant. PAF estimates ranged from 0.9–2.7%, suggesting that up to three percent of adverse birth and infant outcomes could be avoided by eliminating severe housing insecurity among low-income, pregnant women in US cities. **Conclusions:** Results suggest that housing insecurity during pregnancy shapes neonatal and infant health in disadvantaged urban families.

## 1. Introduction

The United States (U.S.) is in the midst of a housing affordability crisis. Because income growth has not kept pace with rising housing costs, fewer and fewer families are able to afford housing [1]. Owing to structural racism in labor and housing markets, Black and Latinx households are disproportionately affected, with single mothers with children facing the highest risk [2,3,4,5,6]. In 2017, a majority of low-income families with children were forced to spend over half of their household income on housing [7]. This affordable housing shortage coincides with increases in evictions and homelessness, two severe forms of housing insecurity [8,9].

Housing insecurity among pregnant women takes a toll on maternal mental health and the health of a fetus. Depression, anxiety, and stress increase a woman’s likelihood of delivering preterm or giving birth to a low birth weight infant [10,11,12]. In a study of low-income, urban mothers, moving two or more times in the past two years was associated with a 1.7 times the odds of depression and a 2.5 times the odds of generalized anxiety disorder, relative to mothers who moved less [13]. In the same population of mothers, eviction was associated with a 21% increase in the probability of depression and a 19% increase in self-reported parenting stress [14]. Subsequently, a number of studies have found housing insecurity and homelessness during pregnancy to be associated with pregnancy complications [15,16], preterm birth, and low birth weight [17,18,19]. Recent studies show that pregnant women living in neighborhoods rendered unstable by evictions and tax foreclosures may be more likely to deliver very low birth weight or preterm infants, particularly if the women have low educational attainment [20,21]. Preterm and low birth weight infants are more likely to require costly intensive care following delivery and may face challenges with physical, cognitive, and social/emotional development later in life [22].

Despite this growing body of evidence, there are important gaps in our knowledge about how housing insecurity in the U.S. may influence the health of infants at birth into childhood. Past studies have not examined the association between housing insecurity on costly birth outcomes such as stays in neonatal intensive care units (NICUs) and extended hospital stays after delivery. Moreover, studies to date have not examined associations between prenatal housing insecurity and children’s health beyond birth. Uncovering the relationship between prenatal housing insecurity on birth-related healthcare utilization and later infant health and development outcomes will add to our understanding of the costs associated with the housing affordability crisis, both human and economic.

To fill these gaps, we conduct analyses testing a hypothesized link between severe housing insecurity (i.e., homelessness or threatened eviction) during pregnancy and adverse health outcomes measured at birth and during infancy in a cohort of low income, urban mothers and infants. Specific outcomes include low birth weight or preterm birth, NICU or stepdown facility stays, and extended hospital stays after delivery, as well as parent-reported health and temperament at age one. Our findings allow stakeholders to gauge population health implications of reducing severe housing insecurity among low-income, pregnant women in U.S. cities. Because evictions and adverse birth outcomes are both concentrated in communities of color, these results have important implications for health equity and social justice.

## 2. Methods

### 2.1. Study Population

We utilized data from the Fragile Families and Child Wellbeing Cohort Study (FFCWS). The study is a birth cohort of nearly 5000 children born to “fragile families” (i.e., disproportionately unmarried, low-income parents) in 20 U.S. cities with populations greater than 200,000 between 1998 and 2000 and followed for 15+ years. The study methods are described in detail in previous publications [23]. Within study cities, live births were randomly selected for participation within strata of marital vs. non-marital births. To be eligible, infants needed two living, English- or Spanish-speaking biological parents. At birth, parents were surveyed for demographic and socioeconomic information, and the study team systematically abstracted information from medical records from the pregnancy and birth, provided that mothers gave consent and hospitals authorized access. The Princeton University Institutional Review Board approved FFCWS study protocols and all participants provided informed consent.

Our study population was comprised of infants for whom maternal medical records were available, who had available information on length of hospital stay and method of delivery, and for whom infant outcomes (i.e., temperament score and parent-rated health) were available at the age one study visit. We excluded infants from multiple gestation pregnancies and those with congenital chromosomal and central nervous system abnormalities because of systematically different birth and infant outcomes in these groups. The Johns Hopkins Bloomberg School of Public Health Institutional Review Board reviewed protocols for this secondary data analysis and determined the project to be non-Human Subjects Research.

### 2.2. Measures

Severe housing insecurity during pregnancy was the main exposure of interest. The binary indicator was abstracted retrospectively from the mother’s medical records and indicates whether there was any mention of “homelessness or threatened eviction” during pregnancy. Because clinicians may have limited knowledge of patients’ housing situation outside of crisis situations, this definition likely fails to capture less severe forms of housing insecurity.

We measured three adverse birth outcomes abstracted from medical records. The first outcome was a composite outcome of low birth weight (<2500 g) and/or preterm birth (<37 weeks gestation) [24]. The second outcome was an indicator of whether the infant stayed in a NICU or intermediate/stepdown facility for any length of time after birth. The third outcome identified infants with extended hospitalization after delivery, defined as greater than two days for vaginally delivered infants or greater than four days for infants delivered by cesarean section [25].

At age one, we measured two infant health outcomes. The first was a measure of infant health, as reported by the infant’s primary caregiver, defined by FFCWS as the biologic parent or adult who lives with the index child at least half of the time, defaulting to the mother if the infant lives with both biologic parents. The caregiver was asked to rate their infant’s general health on a scale as excellent, very good, good, fair, or poor, which we dichotomized to fair or poor health versus excellent, very good, or good. We also constructed a measure of temperament based on three items from the emotionality subscale of the Emotionality, Activity, and Sociability (EAS) Temperament Survey for Children: Parental Ratings [26]. Items included in the scale are “he/she often fusses and cries,” “he/she gets upset easily,” and “he/she reacts strongly when upset,” each of which parents rated from one (“not at all like my child”) to five (“very like my child”). Temperament score at age one is significantly correlated with externalizing behavior at age five among FFCWS participants [27]. Consistent with previous FFCWS analyses using this temperament scale [27,28,29], factor analysis indicated that the three items represented a single factor, with moderate internal consistency (Cronbach’s alpha = 0.60). We summed standardized factor loadings for each of the items to generate a weighted score for temperament with a mean of zero and a standard deviation of approximately one. Infants with temperament scores in the top-most quintile were classified as having a poor temperament.

We included several variables as controls in our analytic models out of concern that they may confound the relationship between severe housing insecurity during pregnancy and birth and infant health outcomes. These variables were largely maternal factors: age group (<20, 20–35, >35), race/ethnicity, poverty level (household income as a percent of the federal poverty level), educational attainment (less than high school, high school or GED, some college, or college degree or above), marital status, pre-pregnancy mental health status, substance use during pregnancy (tobacco, alcohol, or other drugs), and a composite indicator of preexisting conditions (any of the following: hypertension, renal disease, diabetes, lung disease, heart disease, and/or anemia). We also included an indicator for infant sex.

### 2.3. Statistical Analysis

We first compared the distributions of maternal and infant factors as well as birth and infant outcomes by severe housing insecurity during pregnancy, testing for differences with chi square statistics.

**Figure 1:**
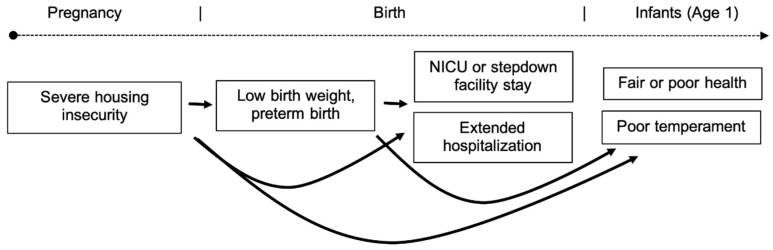
Framework illustrating hypothesized associations between severe housing insecurity (threated eviction or homelessness) during pregnancy, birth outcomes, and infant health and potential pathways linking the exposure and outcomes.

For each birth and infant outcome, we constructed a separate regression model, with housing insecurity as the main exposure. We used Poisson regression with robust variance to approximate log binomial regression models and estimate crude and adjusted risk ratios and 95% confidence intervals for each outcome. We used generalized estimating equations (GEE) to estimate population-averaged associations, accounting for clustering by the infant’s city of birth. For all models of outcomes other than low birth weight and/or preterm birth, we included an indicator for low birth weight and/or prematurity to gauge the degree to which severe housing insecurity acts on outcomes directly vs. indirectly operating indirectly through low birth weight or preterm birth (as specified in our conceptual framework; see Figure 1).

We then estimated the population attributable fraction (PAF) using the formula derived by Miettinen [30] and recommended by Rockhill to produce internally valid estimates of PAF when confounding exists [31]. The formula is as follows:

(1) $$\mathrm{pd}*\frac{\left(\mathrm{aRR}-1\right)}{\mathrm{aRR}}$$

where “pd” indicates the proportion of cases exposed to severe housing insecurity and “aRR” indicating the adjusted risk ratio measuring the association between severe housing insecurity and a given outcome. If we assume that the associations are causal, the PAF can be interpreted as the proportion of outcomes that could be avoided by eliminating severe housing insecurity during pregnancy in the study population.

We conducted all analyses using Stata version 15.1 (StataCorp LLC, College Station, TX, USA), and a *p*-value <0.05 was used to indicate statistical significance. Data were analyzed from 2019–2020.

## 3. Results

**Figure 2:**
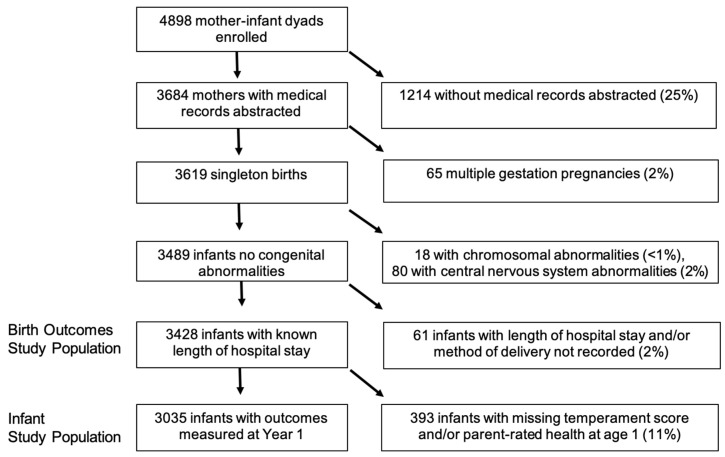
Flow diagram showing specification of study populations sourced from the Fragile Families and Child Wellbeing Study, 1998–2000.

Out of the 4898 mother–infant dyads enrolled in FFCWS, 3428 were included in analyses of birth outcomes, while 3035 were included in analyses of infant outcomes. Figure 2 is flow diagram detailing the specification of the two study populations. Medical records were unavailable for 25% of the source population, chiefly for hospital-level reasons (about two thirds of these women delivered in hospitals that restricted access to patients’ records, or hospitals were unable to locate records despite mothers providing consent, but in one third of cases, because women did not consent to sharing them). We compared characteristics of the source population (i.e., the full FFCWS sample), the birth outcomes study population, and the infant outcomes study population and found no meaningful differences (see Appendix A).

Among the 3428 mother–infant dyads included in the birth outcome analyses, 1.6% had a record of severe housing insecurity during pregnancy (Table 1). Mothers with severe housing insecurity had similar age distributions to other mothers in the sample and their infants were equally likely to be female. On all other factors, the two groups diverged significantly (*p* < 0.05).

**Figure 3:**
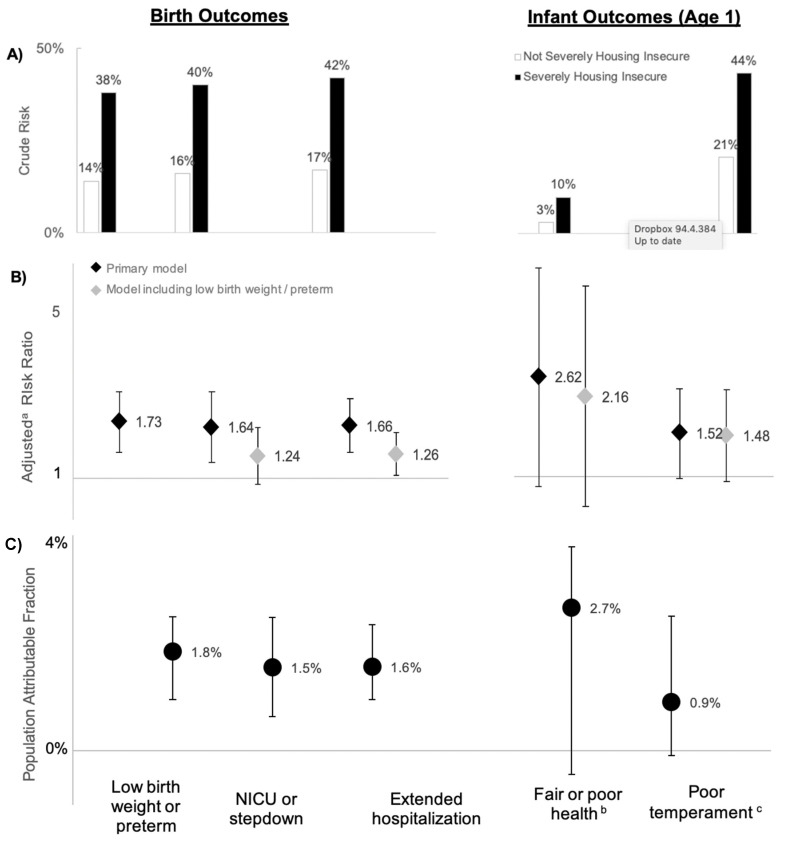
(**A**) Crude risk, (**B**) adjusted risk ratios, and (**C**) population attributable fraction of birth and infant outcomes associated with severe housing insecurity during pregnancy, the Fragile Families and Child Wellbeing Study, 1998–2000. ^a^ Covariates include maternal age category, race/ethnicity, poverty level, education, marital status, pre-pregnancy mental health status, preexisting conditions (hypertension, renal disease, diabetes, lung disease, heart disease, and/or anemia), and infant sex. ^b^ > 2 days for vaginal births, >4 days for cesarean births. ^c^ Scoring in topmost quintile of a scale constructed from the emotionality subscale of the Emotionality, Activity, and Sociability (EAS) Temperament Survey for Children.

The crude risk of each outcome was high in the comparison group and considerably higher among infants born to severely housing insecure mothers. Fourteen percent of infants born to mothers with no reported housing insecurity were born preterm or with low birth weight, 16% stayed in NICUs or stepdown facilities, and 17% had extended hospital stays after delivery. Among infants born to severely housing insecure mothers, these proportions were much higher, at 38, 40, and 42%, respectively. Whereas 3% of infants in the comparison group had fair or poor health at age 1 and 21% had a poor temperament, 10 and 44% of infants in the housing insecure group experienced these adverse outcomes, respectively. Crude risk estimates are depicted in Figure 3A.

In adjusted models, women experiencing severe housing insecurity during pregnancy had 1.73 times the risk of low birth weight and/or preterm birth compared to women who did not experience severe housing insecurity (95% CI 1.28, 2.32; Figure 3B). Severe housing insecurity during pregnancy was also associated with 1.64 times the risk of an infant staying in the NICU or a stepdown facility (95% CI 1.17, 2.31) and 1.66 times the risk of an extended hospitalization following delivery (95% CI 1.28, 2.16). The associations with NICU/stepdown stay and extended hospitalization were attenuated when we added low birth weight or preterm birth to the model, although the risk ratio for extended hospitalization remained statistically significant. Infants born to mothers who experienced severe housing insecurity during pregnancy had 2.62 times the risk of fair or poor health at age one compared to infants born to women with more housing security during pregnancy, although the results were not statistically significant (95% CI 0.91, 7.48). These infants were also 1.52 times as likely as others to have a poor temperament score (95% CI 0.98, 2.34). When we included an indicator for low birth weight and/or preterm birth in models, the risk ratio for fair or poor health was attenuated slightly, whereas the risk ratio for temperament remained largely unchanged.

If these associations are causal, eliminating severe housing insecurity pregnancy may result in the following reductions in negative birth and infant outcomes at the population level: PAF = 1.8% of low birth weight or preterm birth (95% CI 1.0, 2.5); PAF = 1.6% of NICU or stepdown facility stays (95% CI 0.6, 2.5); 1.6% of extended hospital stays after delivery (95% CI 1.0, 2.3); PAF = 2.7% of fair or poor infant health (95% CI −0.4, 3.8); PAF = 0.9% of low infant temperament scores (95% CI −0.1, 2.5) in the study population (Figure 3C). Ninety-five percent confidence intervals for PAF estimates related to infant outcomes (poor infant health and poor temperament) overlapped zero, indicating that the results were not statistically significant.

## 4. Discussion

We tested whether severe housing insecurity (i.e., homelessness or threatened eviction) during pregnancy is linked to adverse child health outcomes measured at birth and age one. In this sample of disproportionately unmarried, low-income mother–child dyads from 20 U.S. cities, there was a 73% higher risk of low birth weight or preterm birth among infants born to mothers who experienced (compared to those who did not) severe housing insecurity during pregnancy. We also found statistically significant increases in the risk of NICU or stepdown stays and extended hospital stays after delivery (64 and 66% higher, respectively) among these dyads. At one year of age, infants of women who experienced severe housing insecurity while pregnant were 2.6 times more likely than others to have fair or poor health and 1.5 times more likely than others to have a poor temperament score, although these differences were not statistically significant. Compared to estimates from primary models, estimates from models including birth outcomes (low birth weight or preterm birth) were, in general, attenuated toward the null. This result is consistent with our hypothesis that some proportion of the associations we see between severe housing insecurity and healthcare and infant outcomes was related to the infant’s preterm or with low birth weight status.

Population attributable fraction estimates suggest that the United States could avoid approximately 1.8% of low birth weight or preterm birth, 1.6% of NICU or stepdown facility stays, 1.6% of extended hospital stays after delivery, 2.7% of fair or poor infant health, and 0.9% of poor infant temperament by eliminating severe housing insecurity among low income, pregnant women in its large cities. While these percentages may seem modest, they represent significant reductions in outcomes that are both common and costly in the U.S., particularly among infants born to disadvantaged women. Nearly 700,000 infants are born to low socioeconomic status, urban mothers each year in the U.S., around fourteen percent of them have low birth weight and/or are preterm [32]. Per our results, if we eliminated severe housing insecurity in this disadvantaged group of mothers, we would expect to see approximately 2000 more term infants born full term and with normal birth weight and, consequently, better health prospects and lower healthcare costs. A 2007 study found that the average preterm infant stays in the NICU for 17.6 days, incurring nearly USD 31,000 in costs (or nearly USD 42,000 in 2019 dollars) [33]. A back-of-the-envelope calculation indicates that these seemingly modest reductions in adverse birth outcomes could translate to USD 84 million in annual savings to the healthcare system and to lower-income families. These savings pertain to birth costs alone, not accounting for savings related to the mother’s health or the infant’s improved health as they grow into childhood, and so are an underestimate of savings across the life course. In fact, research from our group suggests a bi-directional relationship between adverse birth outcomes and housing insecurity, with adverse birth outcomes predicting future eviction risk [34]. As such, promoting housing security during pregnancy may have a multiplicative effect, protecting against future housing insecurity. Future research could use a more rigorous approach to derive a comprehensive estimate of costs stemming from housing insecurity-associated adverse birth outcomes. Such an analysis was beyond the scope of this article.

It should be noted that the women experiencing severe housing insecurity in our sample were likely exposed to multiple social determinants of health concurrently. For example, evidence suggests that people experiencing housing insecurity also struggle to access healthcare [35] and adequate nutrition [36], while also being exposed to higher levels of community violence [37] and environmental toxins [38]. For the purposes of this study, we control for factors that we believe to be “upstream” of housing insecurity, including multiple indicators of socioeconomic status and race/ethnicity as a proxy for experienced racism, but do not delve into co-occurring social determinants of health or specific biologic or social mechanisms through which maternal housing insecurity may lead to adverse birth outcomes.

We believe our estimates to be conservative due to two main data limitations. First, two covariates included in multivariable models could be mediators rather than confounders of the associations due to the time at which they were measured. Maternal poverty and substance use were first measured at birth and during pregnancy, respectively, and thus could represent downstream effects of housing insecurity rather than causes. By including these potential mediators in statistical models, risk ratios estimating associations between severe housing insecurity and outcomes may be biased toward the null, resulting in conservative estimates. Second, measurement error in the exposure could also result in conservative estimates. Clinician notes in the mother’s medical record regarding homelessness or threatened eviction were likely to capture only the most extreme cases of housing insecurity—street homelessness, for example. We expect that a considerable number of women currently considered housing secure in our analysis in fact experienced some degree of housing insecurity during pregnancy. We hypothesize that this misclassification would lead to an excess risk of negative birth and infant outcomes in the comparison group, attenuating estimates. This issue may be compounded in our calculation of population attributable fraction, given that PAFs are a function not only of effect size, but also the prevalence of the exposure, which, again, is likely underestimated in our study population. Housing insecurity has grown dramatically since these data were collected as a consequence of the housing affordability crisis [1]. Currently, in the context of the COVID-19 pandemic and financial crisis, 40% of U.S. renters are struggling to pay rent [39] and 30–40 million people are at risk of eviction [40]. With so many renters facing displacement, the population-level impact of severe housing insecurity on birth and infant outcomes may be larger and more relevant today than ever before.

## 5. Conclusions

Even our conservative estimates suggest that severe housing insecurity during pregnancy contributes to adverse birth and infant outcomes. Because evictions and adverse birth outcomes disproportionately affect Black and Latinx mothers and infants, these results also suggest an opportunity to narrow disparities in birth outcomes. Given the current housing affordability crisis in the U.S., these results warrant attention from clinicians and policymakers. Clinically, our results suggest that prenatal screening and referrals to prevent maternal evictions and homelessness could improve birth and infant outcomes. Thinking more upstream, the results underscore a need for policies to lessen the burden of housing insecurity among pregnant women. Across the country, city and state governments are considering initiatives to increase the stock of affordable housing (e.g., through zoning or subsidized housing) and prevent evictions (e.g., via small grants, legal aid, and eviction moratoria). Our results suggest that pregnant women and their infants stand to benefit greatly from these policy interventions.

## Figures and Tables

**Table 1:** Characteristics of mothers and infants who did vs. did not experience housing insecurity during pregnancy Ϯ (*N* = 3428 mother–infant dyads), the Fragile Families and Child Wellbeing Study, 1998–2000.

No Severe Housing Insecurity during Pregnancy (*N* = 3373)		Severe Housing Insecurity during Pregnancy (*N* = 55)
Maternal Factors (Measured at Birth)		
Age		
<20	623 (18%)	11 (20%)
20–35	2446 (73%)	38 (69%)
≥35	304 (9%)	6 (11%)
Race/ethnicity		
Non-Hispanic white	658 (20%)	6 (11%)
Non-Hispanic Black	1599 (47%)	42 (76%)
Hispanic	973 (29%)	6 (11%)
Other/missing	143 (4%)	1 (2%)
% Federal Poverty		
<50%	637 (19%)	15 (27%)
50–99%	594 (18%)	18 (33%)
100–199%	882 (26%)	16 (29%)
≥200%	1260 (37%)	6 (11%)
Education		
<High school	1220 (36%)	38 (69%)
High school/GED	1022 (30%)	9 (16%)
Some college	796 (24%)	7 (13%)
≥College degree	331 (10%)	1 (2%)
Missing	4 (<1%)	0
Married	790 (23%)	3 (5%)
Pre-pregnancy mental health problems	409 (12%)	35 (64%)
Substance use during pregnancy		
Tobacco	669 (20%)	28 (51%)
Alcohol	248 (7%)	14 (25%)
Drugs	308 (9%)	24 (44%)
Maternal preexisting conditions *	1406 (42%)	35 (64%)
**Infant factors**		
Female sex	1611 (48%)	31 (56%)

* hypertension, renal disease, diabetes, lung disease, heart disease, and/or anemia. Ϯ All *p*-values <0.05 with the exception of maternal age and infant sex.

## Appendix A

**Table A1:** Characteristics of mothers and infants in the source population (i.e., the full Fragile Families and Child Wellbeing baseline sample), the birth outcomes study population, and the infant outcomes study population.

Full Source Population (*N* = 4898)		Birth Outcomes Study Population (*N* = 3428)	Infant Outcomes Study Population (*N* = 3035)
**Maternal factors (measured at birth)**			
Age			
<20	853 (17%)	634 (18%)	583 (19%)
20–35	3585 (73%)	2484 (72%)	2180 (72%)
≥35	460 (9%)	310 (9%)	272 (9%)
Race/ethnicity			
Non-Hispanic white	1030 (21%)	664 (19%)	603 (20%)
Non-Hispanic Black	2326 (47%)	1641 (48%)	1455 (48%)
Hispanic	1336 (27%)	979 (29%)	854 (28%)
Other/missing	206 (4%)	144 (4%)	123 (4%)
% Federal Poverty			
<50%	928 (19%)	652 (19%)	560 (18%)
50–99%	843 (17%)	612 (18%)	534 (18%)
100–199%	1262 (26%)	898 (26%)	799 (26%)
≥200%	1864 (38%)	1266 (37%)	1142 (38%)
Missing	1 (<1%)	0	0
Education			
<High school	1699 (35%)	1258 (37%)	1076 (35%)
High school/GED	1480 (30%)	1031 (30%)	921 (30%)
Some college	1189 (24%)	803 (23%)	733 (24%)
≥College degree	524 (11%)	332 (10%)	303 (10%)
Missing	6 (<1%)	4 (<1%)	2 (<1%)
Married	1187 (24%)	793 (23%)	715 (24%)
Pre-pregnancy mental health problems	--	444 (13%)	376 (12%)
Substance use during			
pregnancy			
Tobacco	--	697 (20%)	599 (20%)
Alcohol	--	262 (8%)	225 (7%)
Drugs	--	332 (10%)	266 (9%)
Maternal preexisting conditions *	--	1441 (42%)	1281 (42%)
**Infant factors**			
Female sex	2341 (48%)	1642 (48%)	1465 (48%)
**Exposure**			
Severe housing insecurity during pregnancy	--	55 (2%)	39 (1%)
**Birth Outcomes**			
Low birth weight or preterm	--	481 (14%)	398 (13%)
NICU stay or stepdown	--	554 (16%)	459 (15%)
Extended hospitalization after delivery	--	586 (17%)	499 (16%)

* hypertension, renal disease, diabetes, lung disease, heart disease, and/or anemia.
